# FTIR typing of the emerging NDM-14-producing *Klebsiella pneumoniae* ST147 clone

**DOI:** 10.1128/jcm.00340-26

**Published:** 2026-08-13

**Authors:** Pablo Aja-Macaya, Lara Díaz Formoso, Tania Blanco-Martín, Lorena López-Cerero, Cristóbal del Rosario-Quintana, Diego García-Martínez de Artola, Germán Bou, Jorge Arca-Suárez, Marina Oviaño

**Affiliations:** 1 Grupo de Investigación en Microbiología. Instituto de Investigación Biomédica A Coruña (INIBIC), Complexo Hospitalario Universitario A Coruña (CHUAC), Sergas, Universidade da Coruña 16737, As Xubias, A Coruña, Spain; 2 Laboratorio de Referencia para tipado molecular y detección de mecanismos de resistencia a antimicrobianos de Andalucía (PIRASOA). Unidad de Gestión Clínica de Microbiología y Enfermedades Infecciosas, Hospital Universitario Virgen Macarena. Instituto de Biomedicina de Sevilla (IBIS), CSIC, Universidad de Sevilla 16778, Seville, Spain; 3 CIBER de Enfermedades Infecciosas (CIBERINFEC), Instituto de Salud Carlos III 38176, Madrid, Spain; 4 Complejo Hospitalario Universitario Insular-Materno Infantil de Gran Canaria, Las Palmas de Gran Canaria, Spain; 5 Hospital Universitario Nuestra Señora de la Candelaria 16825 , Santa Cruz de Tenerife, Spain; National Institute of Allergy and Infectious Diseases Division of Intramural Research, Bethesda, Maryland, USA

**Keywords:** diagnosis, carbapenemase-producing *Klebsiella pneumoniae*, NDM-14, FTIR, clinical microbiology

## Abstract

The emergence and rapid dissemination of NDM-14-producing *Klebsiella pneumoniae* ST147 represents a major challenge for infection control, requiring timely and reliable outbreak detection tools. In this study, we evaluated Fourier-transform infrared (FTIR) spectroscopy as a rapid typing method for outbreak investigation and compared its performance with whole-genome sequencing (WGS). A collection of 64 carbapenemase-producing *K. pneumoniae* isolates, including 30 NDM-14-producing ST147 isolates associated with a regional outbreak in the Canary Islands, was analyzed using FTIR spectroscopy and WGS. FTIR-based clustering was optimized using the polysaccharide spectral region and a customized distance cutoff. Genomic relatedness was assessed using multilocus sequence typing, core-genome single-nucleotide polymorphism (SNP) analysis at multiple thresholds, and clustering agreement indices. FTIR identified a dominant spectral cluster comprising 31 isolates, capturing all outbreak-related isolates with 100% sensitivity and 97% specificity. FTIR clustering showed concordance with genomic outbreak definitions at stringent SNP thresholds (10–18 SNPs), with accuracy exceeding 98%. Pairwise distance analysis revealed low FTIR dissimilarity among closely related isolates, whereas increased dispersion occurred at intermediate genomic distances (15–30 SNPs). Agreement indices showed improved concordance as genomic stringency increased, with the Modified Adjusted Rand Index values reaching 94.75 at the 10-SNP threshold. Importantly, FTIR identified an NDM-14-producing isolate from a distinct clonal background. Overall, FTIR spectroscopy provides a rapid and reliable first-line screening tool for identifying homogeneous outbreak clusters. However, due to lineage-dependent behavior and limited resolution at intermediate genomic distances, WGS remains essential for confirmatory analysis and precise delineation of transmission events.

**IMPORTANCE:**

The rapid spread of multidrug-resistant *Klebsiella pneumoniae* poses a major challenge for infection control, particularly during hospital outbreaks where timely identification of transmission is essential. In this study, we evaluate Fourier-transform infrared (FTIR) spectroscopy as a rapid typing approach and compare its performance with whole-genome sequencing in the context of an outbreak caused by NDM-14-producing *K. pneumoniae* ST147. Our results show that FTIR can reliably identify highly related isolates within a clonal outbreak, supporting early outbreak recognition. However, its performance is influenced by the underlying genomic structure of the population and may require dataset-specific optimization. These findings highlight the potential of FTIR as a first-line screening tool while emphasizing the need for cautious interpretation and integration with genomic methods for accurate outbreak delineation.

## INTRODUCTION

The global spread of *Klebsiella pneumoniae*–producing New Delhi metallo-beta-lactamase (NDM) carbapenemases has become an increasing concern, as highlighted by data from reference laboratories and large-scale surveillance programs such as ATLAS (1). First identified in 2009 in patients with prior healthcare exposure in India (2), NDM-producing Enterobacterales have since disseminated worldwide, particularly across Europe (3), often associated with high-risk *K. pneumoniae* clones such as ST147, ST11, and ST15, and carried on diverse plasmid backbones (4). In Spain, the first NDM-1-producing isolate was reported in 2009 (5), and although national surveillance between 2015 and 2019 showed prevalence rates below 5% (6), recent data from RedLabRA indicate a significant increase, with NDM-producing *K. pneumoniae* accounting for 7.9% of all CPE isolates in 2023 (7).

Among successful *K. pneumoniae* lineages, ST147 stands out as one of the most widely disseminated clones globally (8, 9). Circulating in Spain for over a decade (10), this clone poses a major therapeutic challenge due to its multidrug resistance profile, often co-harboring other carbapenemases such as OXA-48 and KPC (11, 12). Recently, a highly resistant *K. pneumoniae* ST147 clone carrying an NDM variant, NDM-14, has been found to be rapidly disseminating in France. The origin of this clone appears to be related to North Africa, particularly Morocco, where it has been found to be spreading in the community (13, 14). In this context, we have recently reported the cross-border emergence and rapid expansion of this clone in the Canary Islands, Spain, with more than 100 cases of NDM-14-producing *K. pneumoniae* ST147 clone were detected across multiple islands in 2023–2025 (15). In response, genomic and phenotypic surveillance strategies were implemented.

Genomics has established itself as the gold standard for epidemiological typing, owing to its superior discriminatory resolution and reproducible nature. However, it remains relatively expensive and time-consuming, with bottlenecks associated with data processing and analysis, including the need for advanced bioinformatics expertise (16). In contrast, spectroscopic techniques such as Fourier-transform infrared (FTIR) spectroscopy offer a rapid alternative, delivering results in under 3 hours at a lower cost and enabling near-real-time typing in clinical microbiology laboratories (17, 18). The IR Biotyper software is user-friendly and does not require complex bioinformatics pipelines or advanced computational skills, relying instead on integrated analytical workflows. Nevertheless, appropriate methodological optimization and expert interpretation of clustering results remain essential for reliable application.

In the current study, we evaluated the use of FTIR as a rapid typing tool for the identification and discrimination of the NDM-14-producing *Klebsiella pneumoniae* ST147 clone. This approach aims to support outbreak detection and improve our understanding of the epidemiological dynamics of this emerging lineage in clinical settings. Furthermore, given the lack of consensus on the optimal use of FTIR for outbreak investigation (19), we sought to provide practical guidance for the interpretation of FTIR results through systematic comparison with whole-genome sequencing (WGS). By leveraging high-resolution SNP-based genomic data, we establish a benchmarking framework to define the capabilities and limitations of FTIR in outbreak scenarios and to inform its appropriate integration into routine microbiology workflows.

## MATERIALS AND METHODS

### Bacterial isolates

The study included a representative collection of 64 non-duplicate carbapenemase-producing *K. pneumoniae* (CPK) clinical isolates (Table S1). Of these, 17 isolates originated from the Complejo Hospitalario Universitario A Coruña (CHUAC) and were obtained during the continuous surveillance and genomic analysis of CPE in our institution. An additional 16 isolates were part of a collection from the regional Andalusian reference laboratory at Hospital Universitario Virgen Macarena (HUVM). The remaining 31 isolates corresponded to the first non-duplicate NDM-14-producing *K. pneumoniae* strains identified in 2023 through the Network of Diagnostic Laboratories for Healthcare-Associated Infection Surveillance in the Canary Islands. These isolates were collected at the Complejo Hospitalario Universitario Insular Materno Infantil (CHUIMI), located in Las Palmas de Gran Canaria, Gran Canaria, Canary Islands, Spain. For clarity, this will henceforth be referred to as the “Canary Islands outbreak.”

All isolates were identified by Matrix-Assisted Laser Desorption Ionization Time-of-Flight (MALDI-TOF MS) with MALDI Biotyper Smart (Bruker Daltonics, Bremen, Germany). The isolates were screened for carbapenemase production according to the screening cutoff values recommended by the European Committee on Antimicrobial Susceptibility Testing (EUCAST v14.0), that is, with meropenem or ertapenem MICs higher than 0.125 mg/L. Identification was later confirmed by genome sequencing along with confirmation of the resistance mechanisms (15). All isolates were characterized by WGS and FTIR spectroscopy.

### FTIR spectrum acquisition

Isolates were analyzed using the IR Biotyper kit (Bruker Daltonik, Germany) in combination with the IR Biotyper software (OPUS version 8.32, Bruker Daltonik, Germany), following the manufacturer’s instructions. Briefly, bacterial colonies were collected directly from confluent growth on Tryptic Soy Agar (TSA; Becton Dickinson, CA, USA) after 16–20 hours of incubation at 37°C under ambient air conditions and suspended in 50 μL of 70% ethanol in an Eppendorf tube. The suspension was vortexed until homogeneous, and then 50 μL of deionized water was added. Subsequently, 15 μL of the prepared mixture was pipetted onto a silicon target. After complete drying, the target was inserted into the IR Biotyper system. Quality control standards (IRTS1 and IRTS2) were included in each run to ensure measurement accuracy and reproducibility.

Spectra for all samples were acquired in quadruplicate, with each measurement (one spot) requiring approximately 1 minute. Only isolates that yielded at least three out of four satisfactory spectra were included in downstream analyses. Technical replicates were used to ensure measurement reproducibility under conditions representative of routine laboratory workflows. Spectral acquisition was performed in transmission mode (spectral range: 4000–500 cm⁻¹) using OPUS software (Bruker Daltonik, Germany).

### FTIR spectrum analysis

Each spectrum was pre-processed using vector normalization, and the second-order derivative was calculated using the Savitzky–Golay algorithm. For each isolate, an average spectrum of the replicates was computed and used for further analysis. Spectral similarity was evaluated by generating a dendrogram based on hierarchical cluster analysis (HCA), with or without prior application of principal component analysis (PCA) to reduce dimensionality and concentrate the information content. The Euclidean distance metric was used, and clustering was performed using the average linkage method. Analyses were conducted across specific spectral regions associated with major biomolecular components: the membrane lipid region (3,000–2,800 cm⁻¹ and 1,500–1,400 cm⁻¹), the protein region (1,800–1,500 cm⁻¹), and the polysaccharide region (1,300–800 cm⁻¹). These analyses provided a tree-like representation of spectral relationships among isolates.

The IR Biotyper software automatically suggests a cutoff value for cluster definition based on Simpson’s diversity index, where lower cutoff values result in a greater number of defined clusters. FTIR spectra of two or more isolates with a spectral distance equal to or less than the defined cutoff were considered to belong to the same FTIR cluster. To assess the reliability of the clustering, the software calculates the cophenetic correlation coefficient (CCC), which evaluates how well the hierarchical clustering dendrogram preserves the pairwise spectral distances between isolates. CCC values range from 0 to 1, with higher values indicating a better fit between the dendrogram structure and the original distance matrix. This metric provides an independent measure of clustering consistency and differs from Simpson’s diversity index, which reflects the discriminatory power of the method rather than the structural quality of the clustering. In this study, the dendrogram was generated based on FTIR spectral data, while sequence type (ST) information was used to support the interpretation of clustering patterns, with a primary focus on identifying NDM-14-producing *K. pneumoniae* ST147 isolates.

A distance matrix was derived from the dendrogram results by calculating within-group quantiles at a 95% confidence interval. This matrix represents pairwise relationships between spectra according to the distance metric applied in the dendrogram. To facilitate interpretation, distance values were classified based on quantiles: values up to the 90th percentile were defined as *close* and colored red; values exceeding the 100th percentile were defined as *far* and colored blue; and intermediate values remained uncolored (white). Cluster rectangles in the dendrogram delineate regions where spectra are closely grouped according to these distance thresholds.

The distance matrix also included cluster quality criteria, specifically cluster purity and label coherence. By default, both criteria were defined as the isolate ID, under the assumption that replicate measurements and spectra of a given isolate should ideally form a single cluster. Cluster purity and label coherence were expressed as categorical values (*low*, *medium*, and *high*) and were represented by the standard colors orange, yellow, and green, respectively. High purity indicated the presence of a single label (isolate ID) within a cluster, whereas medium and low purity corresponded to the presence of two and more than two labels, respectively.

### Genomic analysis

Isolates from CHUAC and CHUIMI were sequenced in parallel in Illumina NovaSeq6000 (Illumina Inc, CA, United States), and the index case and an additional strain belonging to a different MLST were also sequenced using a PacBio (Pacific Biosciences, CA, United States) platform to obtain high-quality hybrid assemblies and plasmid reconstruction. Isolates from HUVM were only sequenced by Illumina MiSeq.

Assemblies were analyzed using Pathogenwatch (v.23.1.5) (20) and Kleborate (v.3.1.0) (21) to confirm species identification and to characterize antimicrobial resistance genes, virulence-associated determinants, multilocus sequence types (MLST), and core genome MLST (cgMLST) profiles. Genome quality and completeness were assessed using CheckM (v.1.1.3) (22), and only assemblies meeting predefined quality thresholds were included in downstream analyses, including a minimum genome completeness of 97%, a maximum contamination level of 5%, and a mean sequencing depth greater than 30×.

Core and whole-genome single-nucleotide polymorphism (SNP) distances were calculated using Snippy (v.4.6.0) (23), which was also used to generate a core-genome alignment across all isolates. This alignment served as input for phylogenomic reconstruction using FastTree (v.2.1.11) (24) under a generalized time-reversible (GTR) nucleotide substitution model (“-nt -gtr”). The *K. pneumoniae* reference genome TGH13 (CP012745.1, ST147) was used as the mapping reference. The resulting core-genome phylogeny was used to visualize genetic relatedness among isolates and to support genomic cluster definition.

Genomic clusters were defined using complementary approaches, including MLST, cgMLST, and core-genome SNP distance thresholds. For SNP-based clustering, multiple thresholds (200, 150, 100, 50, 30, 15, and 10 SNPs) were evaluated to explore the impact of genetic distance cutoffs on cluster definition. These genomic clusters were compared with clusters derived from FTIR spectroscopy, as described above.

Concordance between genomic clustering methods and FTIR-based clustering was assessed using all pairwise isolate comparisons. For each genomic clustering approach (MLST, cgMLST, and each SNP distance threshold), isolates were classified as belonging to the same or different clusters and compared with FTIR clustering results. True positives (TP) were defined as isolate pairs assigned to the same cluster by both genomic and FTIR methods; true negatives (TN) as pairs assigned to different clusters by both methods; false positives (FP) as pairs grouped together by FTIR but separated by genomic methods; and false negatives (FN) as pairs grouped together genomically but separated by FTIR.

For each genomic clustering approach (MLST, cgMLST, and core-genome SNP thresholds ranging from 200 to 10 SNPs), a separate contingency table was constructed based on all pairwise isolate comparisons, using genomic clustering as the reference standard. These contingency tables formed the basis for calculating sensitivity, specificity, accuracy, positive predictive value (PPV), negative predictive value (NPV), and F1-score for FTIR-based clustering.

In addition to classical performance metrics, overall clustering agreement between genomic methods and FTIR was quantified using the Modified Adjusted Rand Index (MARI) and the Modified Adjusted Wallace Index (MAWI), including the reversed MAWI (25). These metrics range from 0 to 1, with higher values indicating greater agreement between clustering approaches. MARI assesses overall concordance between two clustering partitions, whereas MAWI evaluates the directional agreement, reflecting the probability that isolates grouped together by one method are also grouped together by the other. All metrics were calculated using the mri R package (v.1.0.1) (26). Phylogenetic trees and comparative visualizations were generated using the ggtree (v.3.0.4) (27) and ggplot2 packages in R (28).

## RESULTS

### Description of the isolates

The collection comprised a wide variety of *K. pneumoniae* belonging to 19 different STs and showing wide carbapenemase gene diversity (Table S1). Among the 64 CPK isolates included in the study, 48 produced NDM-type carbapenemases, including 31 NDM-14 producers, 11 NDM-1, 3 NDM-5, 2 NDM-7, and 1 NDM-23. Other metallo-β-lactamases were also represented, including one IMP-8, two VIM-1, and one VIM-33 producers.

Class A carbapenemases were represented by two KPC-2- and one KPC-3-producing isolates, whereas class D carbapenemases were present in seven isolates. Co-production of carbapenemases was observed in two isolates, one carrying NDM-1 plus KPC-2 and the other NDM-5 plus OXA-48.

### FTIR enables rapid outbreak screening of NDM-14-producing *K. pneumoniae* ST147

For FTIR clustering, the best performance was obtained using the polysaccharide spectral region when no dimensionality reduction was applied. Comparative analyses across different spectral regions and preprocessing strategies consistently showed lower clustering resolution for the lipid and protein regions (data not shown). An optimal cutoff value of 0.566 was automatically suggested by the OPUS software based on the entire study data set (*N* = 64; Table S1). However, using this automatic cutoff, our target cluster (the “Canary Islands outbreak”) was not grouped coherently as expected. More specifically, the automatic cutoff resulted in fragmentation of the outbreak-related isolates into multiple smaller clusters and/or singletons, preventing their identification as a single coherent group. This fragmentation would have impaired outbreak recognition, as epidemiologically related isolates would not have been consistently grouped together, thereby limiting the usefulness of FTIR for real-time outbreak detection under default settings. More specifically, the automatic cutoff (0.566) resulted in over-clustering, whereby the main FTIR cluster included not only the 30 outbreak-related isolates but also five additional ST147 isolates (ARGA00051, ARGA00070, AI2830, ARGA00265, and AI2767) that were not epidemiologically related and harbored different resistance mechanisms. This lack of specificity would have impaired outbreak delineation, as unrelated isolates would have been incorrectly grouped within the outbreak cluster, potentially leading to an overestimation of transmission events.

Therefore, we defined a custom cutoff value based on two criteria: first, that independent replicates of the same isolate clustered together such that the majority (>95%) of their spectra were contained within the same cluster; and second, that the “Canary Islands outbreak” was grouped together while minimizing the inclusion of unrelated isolates. Using these criteria, our cutoff was set at 0.165, with a cophenetic correlation coefficient of 0.976, indicating that isolates belonging to the same cluster are very similar and thus supporting the previous adjustment.

**Fig 1:**
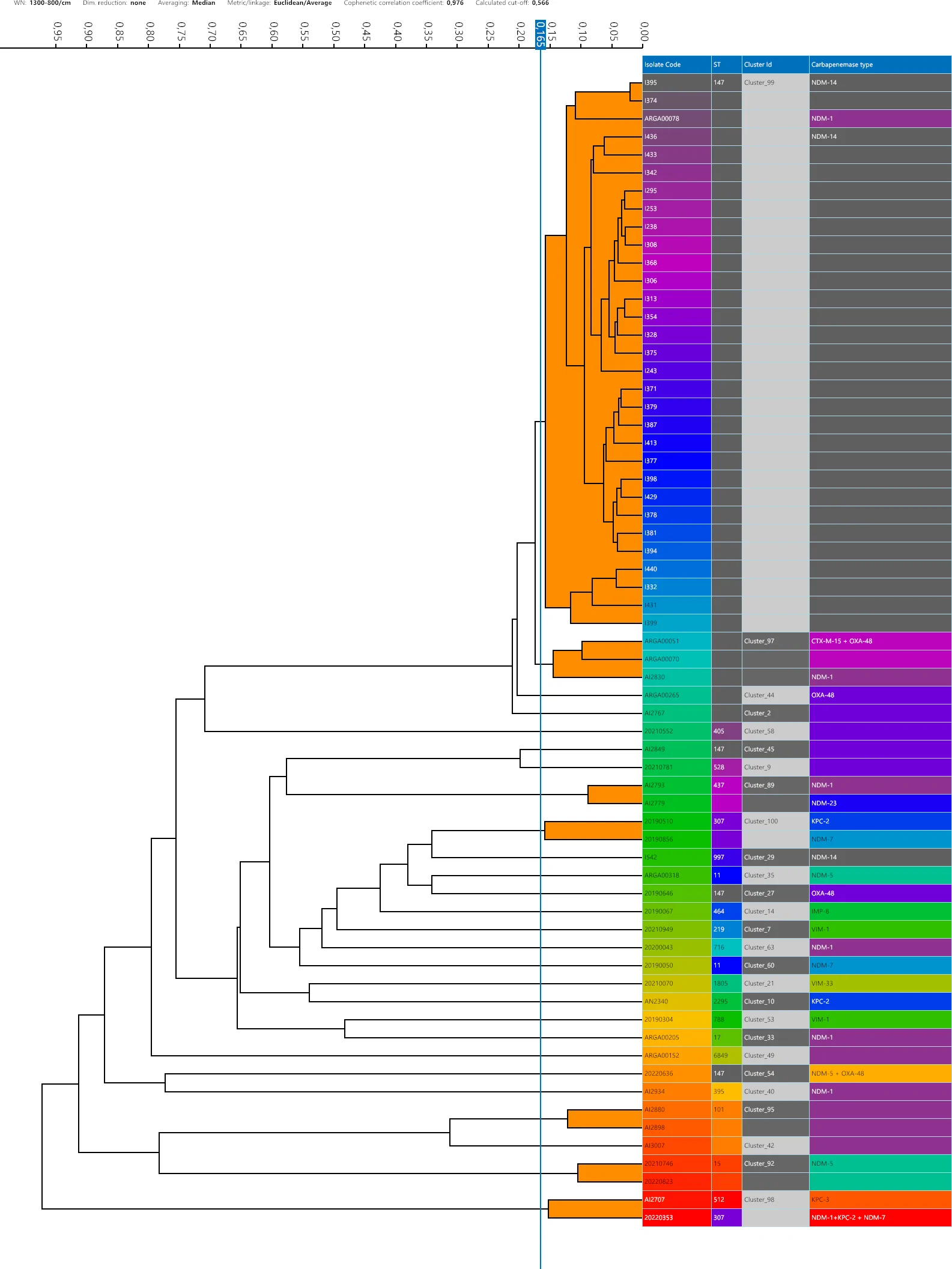
FTIR-based hierarchical clustering of carbapenemase-producing *Klebsiella pneumoniae* isolates included in the outbreak investigation. Hierarchical clustering of 64 carbapenemase-producing *Klebsiella pneumoniae* isolates based on FTIR spectroscopy. Clustering was performed using spectra from the polysaccharide region (1,300–800 cm⁻¹) without dimensionality reduction, applying median averaging and Euclidean distance with average linkage. The dendrogram displays the relative spectral similarity among isolates, with branch lengths representing FTIR distances. The vertical blue line indicates the clustering cutoff applied in this study. Clusters identified by FTIR at this cutoff are highlighted in orange, whereas isolates not grouped into FTIR clusters appear as singletons. The cophenetic correlation coefficient for the dendrogram was 0.976, indicating high internal consistency of the clustering. Colored blocks adjacent to the dendrogram indicate isolate codes, STs, cluster codes, and carbapenemase profiles, as shown in the figure.

Among the 64 CPK isolates tested, FTIR identified 7 clusters comprising 44 isolates and 20 singletons (Fig. 1). The largest cluster (C99) included 31 isolates, of which 30 corresponded to the “Canary Islands outbreak” (100% of outbreak-related isolates), and 1 was unrelated. This yielded a sensitivity of 100% (30/30) and a specificity of 97% (33/34) for the detection of the emerging NDM-14-producing *K. pneumoniae* ST147 clone by FTIR. Additional genomic analyses, including plasmid content (29), CAZyme profiles (30), and antimicrobial resistance determinants, did not identify any features that could explain its clustering with the outbreak group.

Isolates ARGA00051, ARGA00070, and AI2830, also belonging to ST147 (the first two producing OXA-48 and the last producing NDM-1), were grouped into a unique cluster different from C99. In contrast, isolates ARGA00265, AI2767, AI2849, 20190646, and 20220636 from the same ST appeared as singletons.

FTIR clustering also showed concordance with STs for several isolates. Isolates AI2880 and AI2898, both belonging to the ST101 lineage and producing NDM-1, were grouped together, although isolate AI3007, sharing the same characteristics, was not included in this cluster. Similarly, isolates 20220823 and 20210,746 (ST15, NDM-5 producers) were clustered together by FTIR.

In addition, FTIR grouped isolates belonging to the same sequence type despite lacking known epidemiological links, including AI2779 and AI2793 (ST437, producing NDM-23 and NDM-1) and isolates 20190856 and 20190,510 (ST307, producing NDM-7 and KPC-2).

Nevertheless, the established cutoff value also resulted in misclassification in relation to the ST in some cases. For example, isolates 20220353 and AI2707 were grouped together, despite isolate 20220353 being ST307 (producing NDM-1, NDM-7, and KPC-7) and isolate AI2707 being ST512 (producing KPC-3).

Analysis of the distance matrix confirmed “high” label coherence (green), as all technical replicates of the same isolate clustered together, validating the technical reproducibility of the spectral acquisition (Table S2). In contrast, the cluster encompassing the Canary Islands outbreak exhibited “low” purity (orange) due to the presence of 30 distinct isolates within a single spectral group. This low purity value was a direct indicator of the high phenotypic homogeneity among the outbreak isolates, resulting in near-complete spectral overlap and rendering them indistinguishable from one another by FTIR.

Although the distance matrix reflects the same clustering pattern as the dendrogram, it highlights the similarities among the isolates belonging to C99 as well as with five additional ST147 isolates (IDs ARGA00051, ARGA00070, AI2830, ARGA00265, and AI2767), which show slightly higher distance values. Similarly to what is observed in the dendrogram, a small subcluster of four isolates (IDs I399, I431, I332, and I440) can be observed within the main C99 cluster. This subcluster is more distant than the rest of the isolates, with a median distance >0.12, whereas the remaining outbreak isolates display average distances ranging from 0.04 to 0.12.

### Genomic framework for FTIR performance evaluation

#### Comparison of genomic and FTIR-based clustering patterns

**Fig 2:**
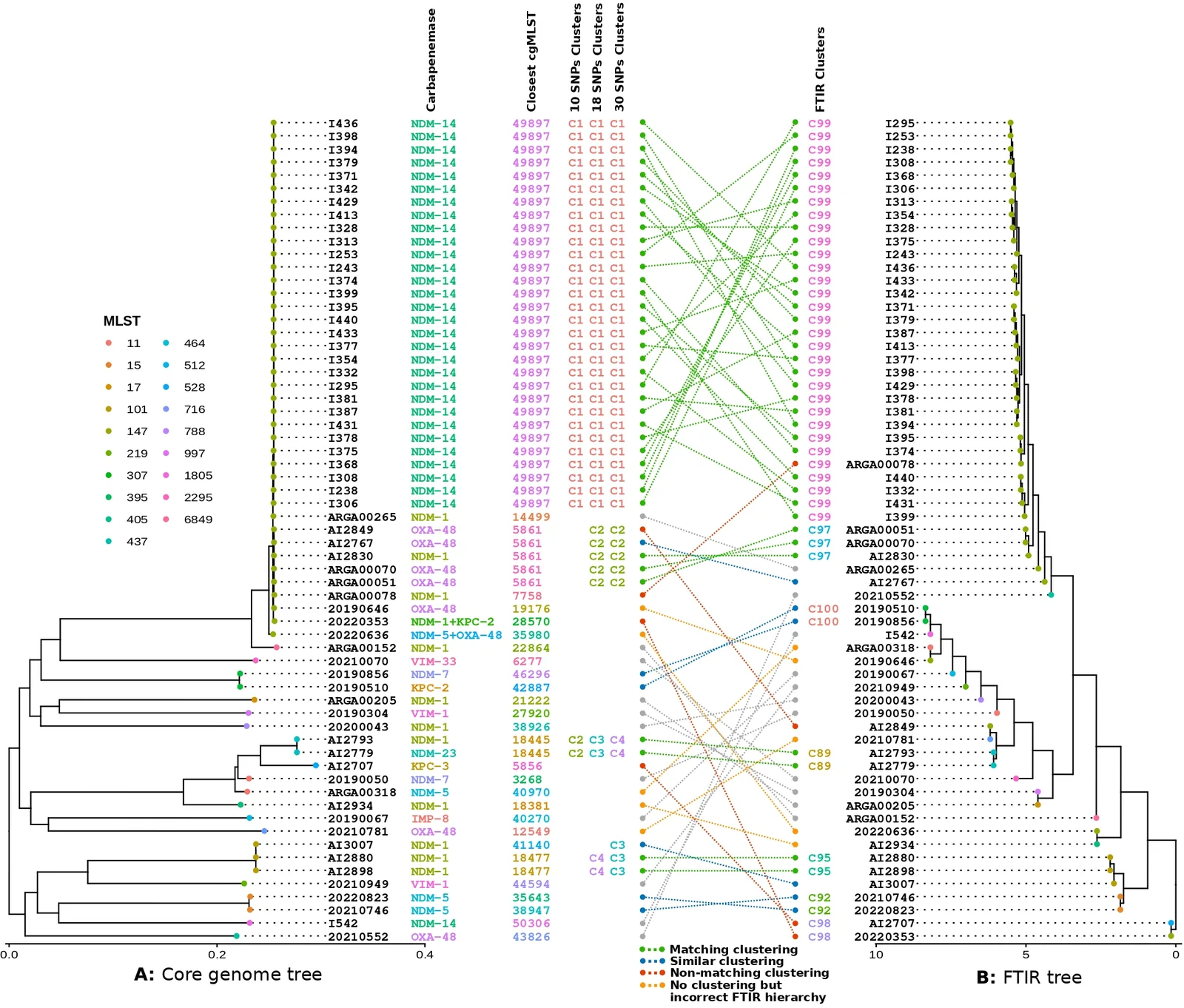
FTIR-based hierarchical clustering compared with core-genome SNP analysis of carbapenemase-producing *Klebsiella pneumoniae* isolates. (**A**) Core-genome SNP–based phylogenetic tree constructed from pairwise core-genome SNP distances, displaying genomic relationships among isolates with clustering visualized at three SNP thresholds (10, 18, and 30 SNPs). (**B**) FTIR-based hierarchical clustering dendrogram based on pairwise FTIR spectral distances, showing the relationships among isolates according to infrared spectral similarity. Isolates are annotated with carbapenemase type, MLST, and cluster assignments derived from FTIR and genomic analyses. The concordance between FTIR- and genome-based clustering is indicated by color coding according to four categories: matching clustering (green), similar clustering (blue), non-matching clustering (red), and no clustering but incorrect FTIR hierarchy (yellow).

The dominant spectral cluster (C99, 31 isolates) obtained by hierarchical clustering based on FTIR spectroscopy largely overlapped with the main genomic outbreak cluster defined by core-genome SNP analysis (C1, 30 isolates), corresponding to the ST147 NDM-14-producing *K. pneumoniae* clone of the Canary Islands outbreak, together with several minor clusters and singletons (Fig. 2).

When applying the most stringent genomic threshold (10 SNPs), the genomic outbreak cluster remained compact and fully contained within the FTIR C99 cluster, corresponding to “matching clustering” between both methods for the core outbreak isolates. At the 18-SNP threshold, which was used as the primary reference for outbreak definition, the genomic cluster remained stable, and FTIR continued to group the majority of these isolates within the same spectral cluster, maintaining a high level of concordance.

At the more permissive threshold of 30 SNPs, the genomic definition of the ST147 outbreak cluster remained unchanged; however, discrepancies between genomic and FTIR clustering became more apparent. While several outbreak-related isolates continued to be grouped within the FTIR C99 cluster, others were assigned to neighboring FTIR clusters, resulting in “similar clustering” rather than strict matching. These discrepancies were confined to isolates at the margins of the outbreak cluster, which showed increased spectral distance from the cluster core and unclear epidemiological linkage.

Finally, isolates that did not belong to the genomic outbreak cluster at any SNP threshold showed either “non-matching clustering” or “no clustering but incorrect FTIR hierarchy,” being separated from the main FTIR cluster or placed in an inconsistent hierarchical position relative to the genomic reference. These patterns were mainly observed among genomically distinct isolates carrying carbapenemases other than NDM-14; however, two ST147 isolates were also positioned by FTIR outside the main outbreak cluster and closer to unrelated sequence types, reflecting phenotypic divergence despite shared clonal background (Fig. 2).

Overall, FTIR reproduced the structure of the main genomic outbreak cluster with high fidelity at SNP thresholds relevant for recent transmission (10–18 SNPs), while divergence between FTIR and genomic clustering increased only when more permissive genomic thresholds were applied.

#### Relationship between core-genome SNP and FTIR distances

**Fig 3:**
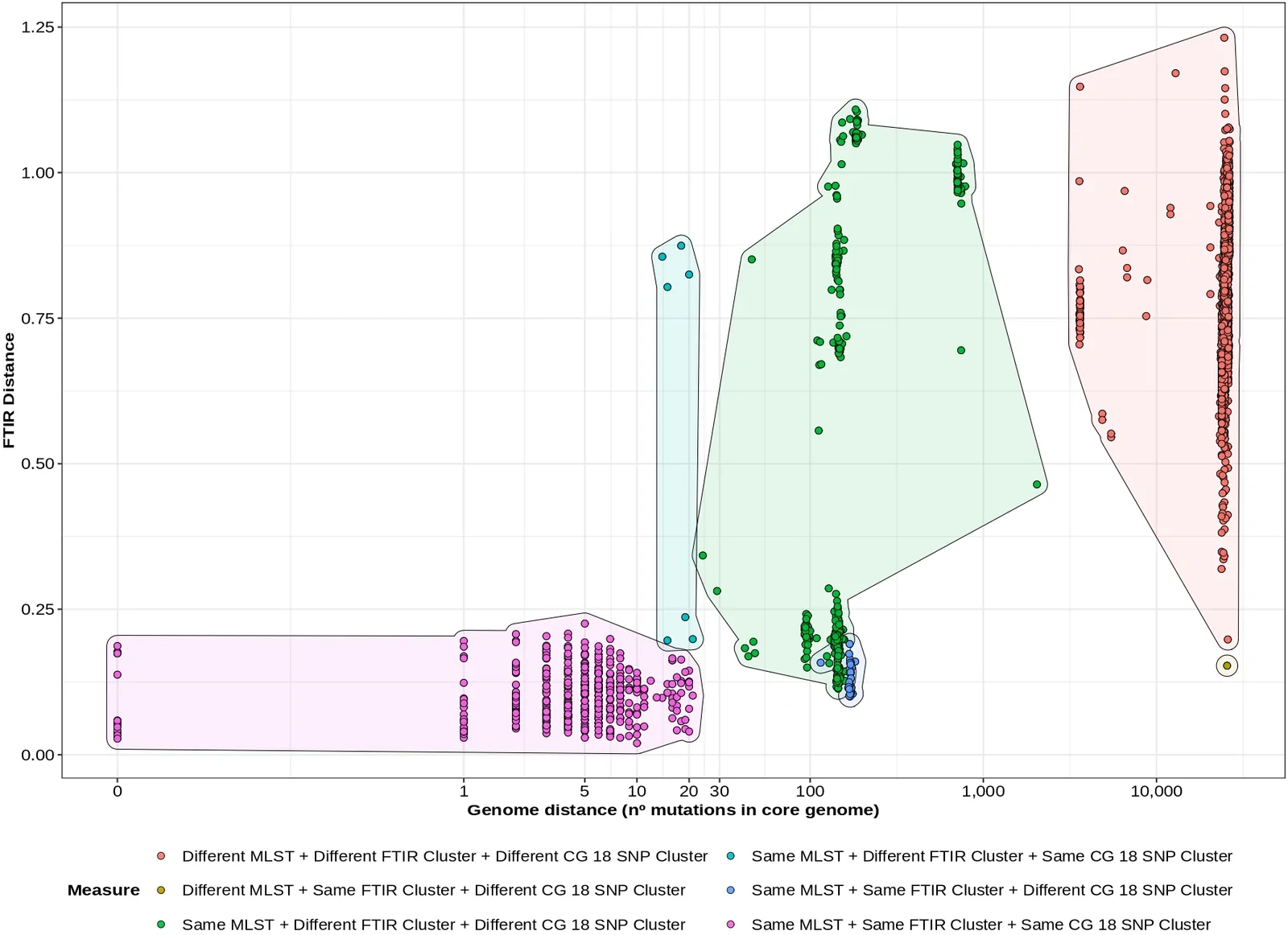
Relationship between core-genome SNP distances and FTIR-based distance values. Pairwise comparison of isolates based on core-genome SNP distances and FTIR-based distance values. Each point represents an isolate pair plotted according to the number of core-genome SNP differences (x-axis) and FTIR distance (y-axis) between them. Isolate pairs are categorized by highlighted regions of color according to the combined outcome of MLST, FTIR clustering, and genomic clustering at the 18-SNP threshold, as indicated in the legend. Categories include isolate pairs sharing the same or different MLST, assigned to the same or different FTIR clusters, and classified as belonging to the same or different genomic cluster.

Analysis of pairwise comparisons showed that isolate pairs belonging to the same genomic cluster defined at the 18-SNP threshold were concentrated at low core-genome SNP distances and low FTIR distance values (Fig. 3). In these pairs, FTIR distance values were consistently below approximately 0.3, with limited dispersion, corresponding predominantly to isolate pairs classified as concordant by both genomic and FTIR clustering.

In contrast, isolate pairs separated by more than 18 core-genome SNPs showed a marked increase in FTIR dispersion. The highest density of discordant FTIR assignments was observed in the intermediate genomic distance range, approximately between 15 and 30 SNPs, where FTIR distance values spanned a broad range, from values comparable to those observed within the outbreak cluster (≤0.3) to values approaching 0.85. This region comprised isolate pairs assigned to different outcome core-genome categories, including pairs grouped together by FTIR despite being genomically distinct, as well as pairs separated by both methods.

At larger genomic distances (>100 SNPs), FTIR distance values were predominantly high, typically above 0.6, and isolate pairs were consistently separated by FTIR and genomic clustering. Although occasional discordant FTIR assignments were still observed in this range, they were less frequent than in the intermediate genomic distance region and mainly reflected incorrect FTIR hierarchical placement rather than clustering overlap. These comparisons largely corresponded to discordant or true-negative classifications and contributed to the high specificity observed in the performance analysis.

#### Effect of genomic clustering stringency on FTIR performance

**Fig 4:**
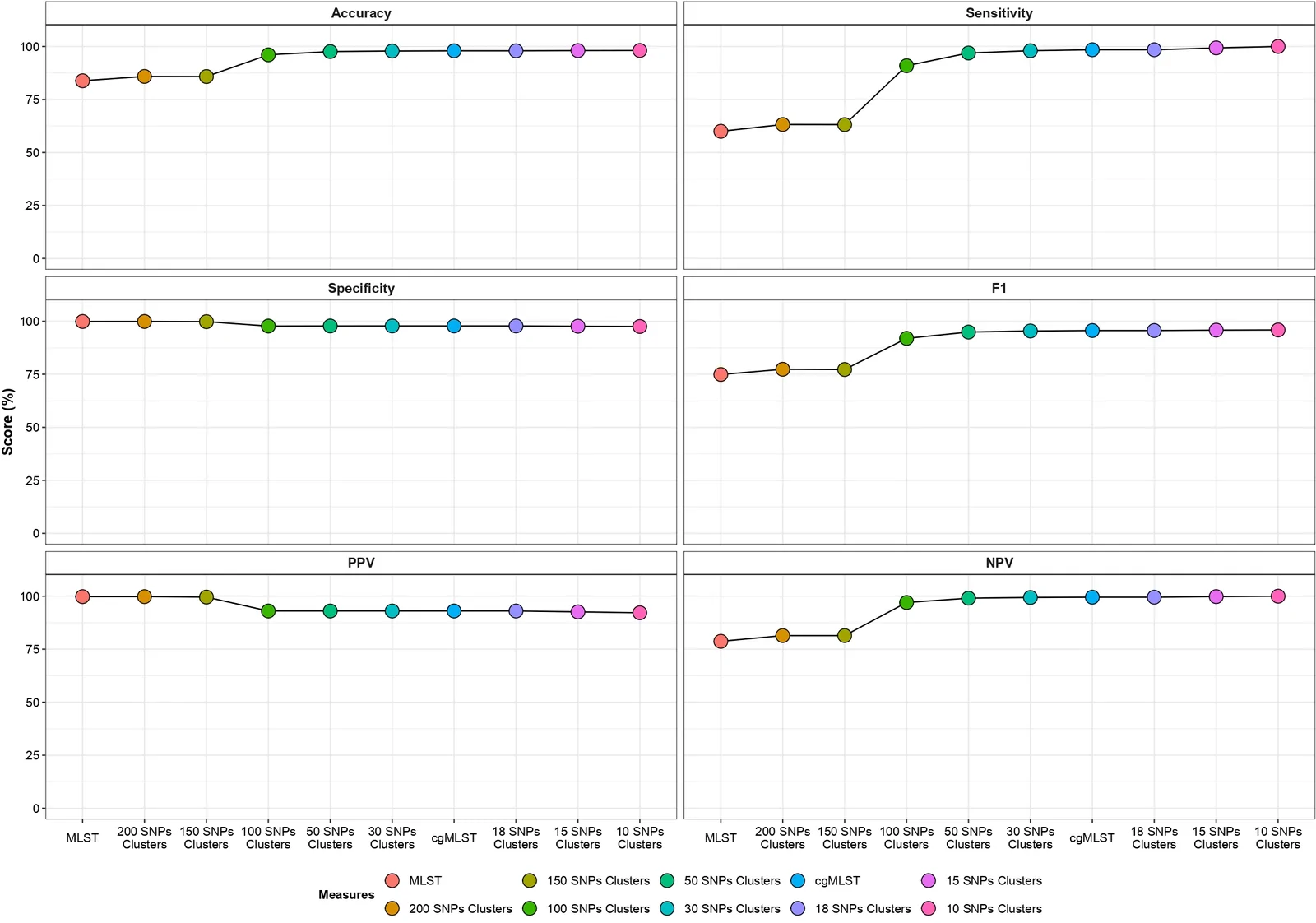
Performance metrics of FTIR-based clustering using genomic clustering as a reference. Performance of FTIR-based clustering compared with genomic clustering used as reference. Sensitivity, specificity, accuracy, PPV, NPV, and F1 score were calculated from pairwise isolate comparisons for different genomic clustering approaches, including MLST, core-genome SNP thresholds (200, 150, 100, 50, 30, 15, and 10 SNPs), and cgMLST-based clustering at the 18-SNP threshold. Bars represent the percentage values obtained for each performance metric. Metrics were derived from contingency tables constructed for each genomic clustering approach. PPV and NPV are included as part of the performance metrics. As these measures are prevalence-dependent, the values reported here reflect the specific epidemiological context of the outbreak-derived isolate collection and should not be extrapolated to other clinical settings.

Analysis of FTIR performance across different genomic clustering thresholds showed a clear trend toward improved concordance, with more stringent SNP cutoffs. Using the 18-SNP threshold as the genomic reference, FTIR achieved a sensitivity of approximately 98.4% and an overall accuracy of 98.0% while maintaining a specificity above 97.8% (Fig. 4; Table S3). When a more stringent threshold of 10 SNPs was applied, sensitivity increased further, reaching even 100%, whereas specificity remained consistently high (>97%).

This trend was consistent with the distribution of pairwise distances observed in Fig. 3. Isolate pairs within the most stringent genomic clusters (≤10–18 SNPs) were associated with low FTIR distance values and limited dispersion, resulting in a low number of false-negative FTIR assignments. In contrast, when more permissive genomic thresholds were applied (≥30 SNPs), additional isolate pairs with heterogeneous FTIR distance values were included, leading to an increase in false-positive assignments and a corresponding reduction in positive predictive value, while sensitivity was only marginally affected.

Overall accuracy remained stable across thresholds, reflecting the predominance of true-negative comparisons and the consistent separation of genomically distant isolates by FTIR. These results indicate that FTIR performance is highest when applied to the identification of highly homogeneous genomic clusters and decreases progressively as genomic clustering criteria are relaxed.

#### Accuracy of FTIR clustering based on contingency table analysis

Contingency table analysis further highlighted substantial differences in FTIR performance depending on the reference clustering method used (Table S4). When MLST was used as reference, overall accuracy values varied across sequence types. In the predominant lineage ST147 (*n* = 40), FTIR achieved an accuracy of 77.5%, reflecting partial dispersion of isolates from this sequence type across multiple FTIR clusters. Lower accuracy values were observed for ST101 (*n* = 3; 66.7%) and ST11 (*n* = 2; 50%); however, these observations should be interpreted with caution due to the very small number of isolates available for these sequence types.

In contrast, when genomic clustering based on the 18-SNP threshold was used as reference, FTIR accuracy increased markedly and became more consistent across clusters. The main genomic outbreak cluster (C1; *n* = 30) was fully captured by FTIR, with all C1 isolates grouped within the same FTIR cluster (C99), corresponding to 100% concordance from a genomic-to-FTIR perspective. However, the FTIR cluster C99 comprised one additional isolate not belonging to C1, resulting in an accuracy of 96.77% when assessed from an FTIR-to-genomic perspective.

Lower accuracy values were restricted to minor genomic clusters, such as C2 (*n* = 5; 60%). In contrast, all remaining genomic clusters and singletons (*n* = 29) were fully contained within a single FTIR cluster (100%), although some FTIR clusters included isolates belonging to different genomic clusters. Overall, these results indicate that FTIR clustering aligns substantially better with genomic outbreak definitions than with MLST-based grouping, consistent with the higher agreement indices observed for SNP-based clustering approaches.

#### Clustering agreement between FTIR and genomic typing methods using agreement indices

**Fig 5:**
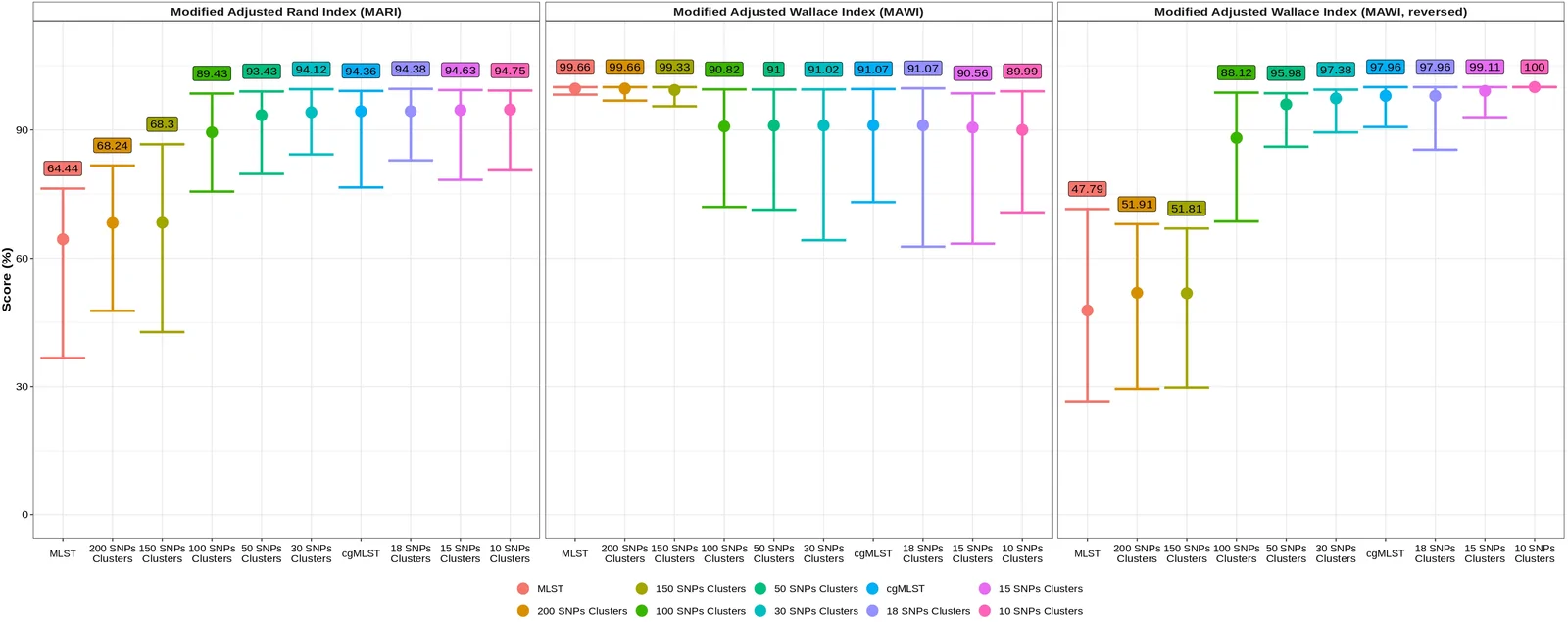
Clustering agreement between FTIR-based typing and genomic approaches assessed by MARI, MAWI, and reverse MAWI across different genomic clustering thresholds. Agreement between FTIR-based clustering and genomic typing methods was evaluated using the MARI, the MAWI, and its reverse formulation (reverse MAWI) across different genomic clustering thresholds. MARI represents overall clustering agreement, MAWI represents the directional agreement from FTIR to genomic clustering, and reverse MAWI represents the directional agreement from genomic to FTIR clustering. Analyses were performed using MLST and SNP-based genomic clustering at increasing levels of stringency. Exact index values are provided in Table S5.

Clustering agreement between FTIR-based clustering and genomic typing methods was quantified using the MARI and the MAWI, together with its reverse formulation (Fig. 5; Table S5). When MLST was used as the genomic reference, overall agreement with FTIR was limited, with a MARI value of 64.44, indicating poor concordance for closely related isolates. Application of core-genome-based approaches resulted in a marked increase in clustering agreement. Using the 18-SNP threshold as a genomic reference, FTIR achieved a MARI value of 92.31, while the maximum overall agreement was observed at the most stringent genomic definition (10 SNPs), where MARI reached 94.75.

Directional agreement analysis revealed clear differences between MAWI and reverse MAWI across typing strategies (Fig. 5). MAWI values were highest when MLST was used as the reference; however, this apparent agreement reflects the limited discriminatory resolution of MLST rather than true biological concordance. When whole-genome SNP-based clustering was applied, MAWI values decreased and showed greater dispersion as genomic resolution increased, indicating that FTIR clustering does not consistently reproduce highly fine-grained genomic cluster definitions.

In contrast, reverse MAWI values were low when low-resolution genomic typing was used. With MLST as the reference, reverse MAWI was 47.79 with wide dispersion. Only minor increases were observed at permissive genomic thresholds (200–150 SNPs), whereas a marked rise occurred at the 100-SNP threshold (88.12), after which reverse MAWI remained high at more stringent cutoffs. This pattern indicates that FTIR clustering begins to reliably reproduce genomically defined clusters when isolates belong to closely related genomic groups.

Together, these results indicate that overall agreement (MARI) and directional agreement indices capture complementary aspects of concordance between FTIR and genomic clustering. While stringent SNP thresholds maximize overall clustering agreement, the sharp increase in reverse MAWI at intermediate genomic thresholds highlights the point at which FTIR begins to consistently recapitulate genomically defined clusters.

## DISCUSSION

In this study, we evaluated the performance of FTIR spectroscopy as a rapid typing tool during the investigation of an outbreak caused by the emerging NDM-14-producing *K. pneumoniae* ST147 clone. By systematically comparing FTIR-based clustering with multiple genomic typing frameworks, we aimed to define the strengths, limitations, and optimal use of FTIR in relation to whole-genome–based approaches, with particular emphasis on high-resolution benchmarking against WGS, to better position its potential role in outbreak investigation. Importantly, the use of WGS at SNP-level resolution in this study was intentional and represents a key strength of our approach. Rather than reflecting a dependency of FTIR on genomic data, this design allowed us to systematically define the conditions under which FTIR clustering is reliable. This comparative framework provides practical guidance for interpreting FTIR results in real-world settings where genomic data may not be immediately available.

Beyond clustering agreement metrics, several methodological and spectroscopic aspects of the FTIR analysis support its value as a rapid typing tool. Optimal discrimination was achieved using the polysaccharide spectral region without dimensionality reduction, highlighting the relevance of surface-associated structures, such as capsular polysaccharides, in the phenotypic differentiation of closely related *K. pneumoniae* isolates (Fig. 1) (31). This observation is consistent with the clonal nature of the ST147 lineage, in which capsule composition is expected to remain highly conserved during early transmission events, as reflected by the low intra-cluster FTIR distances observed among outbreak-related isolates (32). Although the polysaccharide region provided the best performance in this study and is commonly reported as the most informative region for *K. pneumoniae* (17, 18), the optimal spectral region may vary depending on the organism, lineage, or outbreak context. Further studies are needed to determine the extent to which these findings can be generalized across different bacterial populations.

The very high cophenetic correlation coefficient obtained (0.976) indicates strong internal consistency of the FTIR-based clustering, demonstrating that the dendrogram accurately preserves the underlying spectral distance relationships. This supports the robustness of the selected analytical parameters and suggests that the observed clustering structure is driven by genuine phenotypic similarities rather than analytical noise or methodological artifacts.

An additional methodological limitation relevant to routine clinical implementation concerns the definition of the FTIR clustering cutoff. In this study, the cutoff value automatically suggested by the software did not yield biologically coherent clustering of outbreak-related isolates, as it resulted in over-clustering, whereby the main FTIR cluster included not only outbreak-related isolates but also additional ST147 isolates that were not epidemiologically linked and carried different resistance mechanisms. This lack of specificity would have impaired accurate outbreak delineation by overestimating the extent of transmission.

To address this, a customized cutoff was defined based on technical replicates and epidemiological coherence. This approach was intended to ensure epidemiologically meaningful clustering rather than to optimize performance metrics. While this optimization substantially improved clustering performance, it also highlights that FTIR-based typing may require expert optimization and interpretation, limiting its straightforward implementation in routine settings and underscoring the need for standardized guidance (33). Without such standardization, FTIR-based typing may require case-by-case optimization, which could limit its practicality for routine implementation. Furthermore, such customization may require the inclusion of a sufficiently representative isolate collection within each analysis, which could complicate the interpretation of unrelated isolates.

Low intra-cluster FTIR distance values observed among outbreak-related isolates reflect a high degree of phenotypic homogeneity, consistent with the compact genomic structure observed at stringent SNP thresholds. Within this overall homogeneous cluster, however, a small subset of isolates displayed moderately increased FTIR distances, indicating early divergence despite close genomic relatedness (Fig. 2). Most outbreak-related isolates showed very low FTIR distances (0.04–0.12), whereas a small internal subcluster exhibited higher median values (>0.12) while remaining genomically related. This intracluster heterogeneity highlights the limits of FTIR discrimination at fine scales and provides a mechanistic explanation for the reduced predictive value observed in isolates located at the margins of the outbreak cluster. Importantly, this pattern does not necessarily reflect misclassification; rather, it underscores the sensitivity of FTIR to subtle variation that may precede clear genomic separation. Notably, no distinct temporal pattern was observed among these isolates, suggesting that the observed phenotypic divergence is unlikely to be driven by the timing of isolate collection (15). Similarly, no consistent differences were identified in plasmid content (29) or CAZyme profiles (30), further supporting that these variations are not explained by the genomic features analyzed.

This intracluster phenotypic heterogeneity provides an essential framework for interpreting FTIR performance across different genomic clustering thresholds. Although FTIR provides lower discriminatory resolution than whole-genome sequencing, its performance improved when increasingly stringent genomic thresholds were applied, as shown by the higher concordance and performance metrics observed at the 10–18 SNP thresholds (Fig. 3 and 4). This apparent paradox reflects the fact that FTIR captures global biochemical features that remain conserved among closely related isolates but may diverge before substantial genomic distance accumulates. Consequently, FTIR is particularly well-suited for the rapid identification of highly homogeneous outbreak clusters, but less reliable for discriminating isolates at intermediate levels of genomic divergence, where increased dispersion of FTIR distances and reduced predictive values were observed (Fig. 3). These findings may not be directly transferable to organisms with highly dynamic genomic structures or increased phenotypic variability, where FTIR-based discrimination could be further challenged.

An additional observation further illustrates the specificity of FTIR-based clustering. Isolate I542, which carried the same carbapenemase variant (NDM-14) but belonged to a different clonal background and emerged in temporal association with the Canary Islands outbreak, was correctly excluded from the main FTIR outbreak cluster. This indicates that FTIR discrimination is not driven by the resistance mechanism itself, but rather by phenotypic features linked to clonal background. Conversely, the inclusion of a single unrelated isolate within the main FTIR cluster, for which no clear genomic explanation was identified, suggests that FTIR may capture subtle similarities not fully resolved by current genomic markers, consistent with previous observations that FTIR reflects global phenotypic variation associated with bacterial adaptation and clonal evolution (34). Together, these findings highlight both the specificity and the limits of FTIR as a phenotypic typing approach, supporting its use for clonal discrimination rather than for detecting resistance determinants. Accordingly, FTIR results should be interpreted alongside antimicrobial susceptibility testing and molecular data. Further investigation of such discordant cases would require larger collections of isolates within the same sequence type to better characterize within-lineage variability and its genomic basis.

MARI, MAWI, and reverse MAWI showed distinct but complementary trends across genomic clustering approaches (Fig. 5). MARI values increased with genomic resolution and reached their highest values at the most stringent SNP thresholds, reflecting improved overall concordance between FTIR and genomic clustering. In contrast, directional indices revealed asymmetric patterns of agreement.

MAWI values were consistently high across clustering approaches, indicating that isolates grouped together by FTIR were frequently genomically related, but they did not increase monotonically with increasing SNP stringency and showed greater dispersion at stricter thresholds. Conversely, reverse MAWI values were low and highly variable when low-resolution genomic methods were used, followed by a marked increase at the 100-SNP threshold. This transition indicates that consistent FTIR grouping of genomically defined clusters emerges as genomic relatedness increases.

When considered as a screening tool for outbreak investigation, FTIR is more likely to overestimate relatedness rather than underestimate it, particularly when applied beyond highly homogeneous genomic clusters. Our results indicate that FTIR rarely groups together genomically distant isolates, supporting its high specificity and low risk of falsely merging unrelated transmission events. However, in the intermediate genomic distance range, FTIR may group isolates that are no longer part of the same transmission chain, leading to an apparent expansion of outbreak clusters.

From a practical standpoint, this behavior is advantageous for initial outbreak detection, as it favors sensitivity and minimizes the risk of missing related cases. In routine settings, FTIR can be used as a rapid first-line screening tool to identify potentially related isolates and provide an initial clustering framework, thereby guiding the need for confirmatory whole-genome sequencing. In practice, where infection prevention measures are often triggered by epidemiological signals before detailed typing data are available, the same-day turnaround of FTIR may provide early insight into isolate relatedness, supporting decisions on whether to maintain or adjust control measures and helping to prioritize isolates for confirmatory genomic analysis. Accordingly, FTIR-based clustering may be sufficient to exclude clearly unrelated isolates and to rapidly flag clusters compatible with recent transmission, while whole-genome sequencing remains essential for isolates at the margins of FTIR-defined clusters or lacking clear epidemiological links. Importantly, FTIR distance thresholds may not be directly transferable and require context-specific calibration, as clustering behavior is influenced by the population structure and phenotypic stability of the lineage under investigation. In this context, our results provide a practical framework for interpreting FTIR clustering in outbreak scenarios.

A further limitation of this study is the relatively small number of isolates analyzed, which may introduce a degree of selection bias. However, the study was specifically designed to evaluate FTIR performance in the context of a well-defined outbreak with a high a priori probability of clonal relatedness, rather than in a broad surveillance setting. Therefore, the high level of concordance observed should be interpreted within this framework, as it likely reflects the behavior of FTIR in highly homogeneous populations. In contrast, studies, including more heterogeneous and epidemiologically diverse isolate collections, have reported lower concordance values, highlighting the impact of population structure on FTIR performance (17).

In this context, a combined FTIR-WGS approach allows FTIR to be used as an efficient first-line screening tool, while reserving whole-genome sequencing for isolates requiring higher-resolution confirmation. By systematically benchmarking FTIR performance against SNP-level genomic data, this study provides a practical framework for interpreting FTIR clustering in clinically relevant outbreak scenarios and contributes to defining its role within routine microbiology workflows. While high-resolution outbreak analyses may be limited in certain routine hospital laboratories, FTIR may provide value as a decentralized screening tool, enabling rapid local assessment of isolate relatedness and supporting more targeted referral for genomic analysis. As genomic surveillance becomes increasingly integrated into routine clinical practice, the role of rapid complementary tools such as FTIR may become more relevant.

## Data Availability

The whole-genome sequencing data generated in this study have been deposited in the NCBI database under multiple BioProject accession numbers (PRJEB39112, PRJEB42440, PRJEB53686, PRJEB53700, PRJEB70314, PRJNA1104021, PRJNA1133624, and PRJNA1216752). Detailed isolate identifiers and corresponding accession numbers are provided in Table S1. All other data supporting the findings of this study are available from the corresponding author upon reasonable request.

## SUPPLEMENTAL MATERIAL

Table S1 jcm.00340-26-s0001.xlsx Overview of *Klebsiella pneumoniae* isolates analyzed, including sequence type, carbapenemase content, genomic metadata, and clustering information obtained by FTIR and genome analysis.

Table S2 jcm.00340-26-s0002.xlsx Heat map overview for FTIR-based clustering.

Table S3 jcm.00340-26-s0003.xlsx Performance metrics of FTIR in comparison with genomics.

Table S4 jcm.00340-26-s0004.xlsx Contingency tables based on pairwise isolate comparisons, using genomic clustering as the reference standard.

Table S5 jcm.00340-26-s0005.xlsx Pairwise comparison of clustering partitions based on FTIR, MLST, and whole-genome SNP thresholds using agreement indices.

ASM does not own the copyrights to Supplemental Material that may be linked to, or accessed through, an article. The authors have granted ASM a non-exclusive, world-wide license to publish the Supplemental Material files. Please contact the corresponding author directly for reuse.

## References

[1] Wise MG, Karlowsky JA, Mohamed N, Hermsen ED, Kamat S, Townsend A, Brink A, Soriano A, Paterson DL, Moore LSP, Sahm DF. 2024. Global trends in carbapenem- and difficult-to-treat-resistance among World Health Organization priority bacterial pathogens: ATLAS surveillance program 2018-2022. J Glob Antimicrob Resist 37:168–175. doi:10.1016/j.jgar.2024.03.020 38608936

[2] Kumarasamy KK, Toleman MA, Walsh TR, Bagaria J, Butt F, Balakrishnan R, Chaudhary U, Doumith M, Giske CG, Irfan S, et al.. 2010. Emergence of a new antibiotic resistance mechanism in India, Pakistan, and the UK: a molecular, biological, and epidemiological study. Lancet Infect Dis 10:597–602. doi:10.1016/S1473-3099(10)70143-2 20705517 PMC2933358

[3] Johnson AP, Woodford N. 2013. Global spread of antibiotic resistance: the example of New Delhi metallo-β-lactamase (NDM)-mediated carbapenem resistance. J Med Microbiol 62:499–513. doi:10.1099/jmm.0.052555-0 23329317

[4] Pérez-Vázquez M, Sola Campoy PJ, Ortega A, Bautista V, Monzón S, Ruiz-Carrascoso G, Mingorance J, González-Barberá EM, Gimeno C, Aracil B, Sáez D, Lara N, Fernández S, González-López JJ, Campos J, Kingsley RA, Dougan G, Oteo-Iglesias J, Spanish NDM Study Group. 2019. Emergence of NDM-producing *Klebsiella pneumoniae* and *Escherichia coli* in Spain: phylogeny, resistome, virulence and plasmids encoding *bla*_NDM_-like genes as determined by WGS. J Antimicrob Chemother 74:3489–3496. doi:10.1093/jac/dkz366 31504589

[5] Oteo J, Domingo-García D, Fernández-Romero S, Saez D, Guiu A, Cuevas O, Lopez-Brea M, Campos J. 2012. Abdominal abscess due to NDM-1-producing *Klebsiella pneumoniae* in Spain. J Med Microbiol 61:864–867. doi:10.1099/jmm.0.039495-0 22383442

[6] Cañada-García JE, Moure Z, Sola-Campoy PJ, Delgado-Valverde M, Cano ME, Gijón D, González M, Gracia-Ahufinger I, Larrosa N, Mulet X, et al.. 2022. CARB-ES-19 multicenter study of carbapenemase-producing *Klebsiella pneumonia*e and *Escherichia coli* from all spanish provinces reveals interregional spread of high-risk clones such as ST307/OXA-48 and ST512/KPC-3. Front Microbiol 13:918362. doi:10.3389/fmicb.2022.918362 35847090 PMC9279682

[7] Cañada-García JE. 2025 SO-01-0009-RedLabRA: vigilancia molecular de Klebsiella pneumoniae, *Escherichia coli* y Enterobacter cloacae complex productores de carbapenemasas en España en 2023. *In* XXVIII National Congress of the Spanish Society of Infectious Diseases and Clinical Microbiology. Málaga, Spain.

[8] Peirano G, Chen L, Kreiswirth BN, Pitout JDD. 2020. Emerging antimicrobial-resistant high-risk *Klebsiella pneumoniae* Clones ST307 and ST147. Antimicrob Agents Chemother 64:e01148-20. doi:10.1128/AAC.01148-20 32747358 PMC7508593

[9] Rodrigues C, Desai S, Passet V, Gajjar D, Brisse S. 2022. Genomic evolution of the globally disseminated multidrug-resistant *Klebsiella pneumoniae* clonal group 147. Microb Genom 8:000737. doi:10.1099/mgen.0.000737 35019836 PMC8914359

[10] Arca-Suárez J, Rodiño-Janeiro BK, Pérez A, Guijarro-Sánchez P, Vázquez-Ucha JC, Cruz F, Gómez-Garrido J, Alioto TS, Álvarez-Tejado M, Gut M, Gut I, Oviaño M, Beceiro A, Bou G, GEMARA-SEIMC/REIPI Enterobacterales Study Group. 2022. Emergence of 16S rRNA methyltransferases among carbapenemase-producing Enterobacterales in Spain studied by whole-genome sequencing. Int J Antimicrob Agents 59:106456. doi:10.1016/j.ijantimicag.2021.106456 34688835

[11] Sandfort M, Hans JB, Fischer MA, Reichert F, Cremanns M, Eisfeld J. 2022. Increase in NDM-1 and NDM-1/OXA-48-producing *Klebsiella pneumoniae* in Germany associated with the war in Ukraine, 2022. Euro Surveill 27. doi:10.2807/1560-7917.ES.2022.27.50.2200895 PMC9808319 36695468

[12] Rigatou A, Afolayan AO, Tatsi E-B, Deliolanis I, Michos A, Reuter S, Daikos GL. 2025. Double carbapenemases in *Klebsiella pneumoniae* blood isolates: dissemination in a single medical center via multiple plasmids and a variety of highly efficient clones. Antimicrob Agents Chemother 69:e0146224. doi:10.1128/aac.01462-24 39898665 PMC11881573

[13] Emeraud C, Mahamat A, Jousset AB, Bernabeu S, Goncalves T, Pommier C, Girlich D, Birer A, Rodriguez C, Pawlotsky J-M, Naas T, Bonnin RA, Dortet L. 2023. Emergence and rapid dissemination of highly resistant NDM-14-producing *Klebsiella pneumoniae* ST147, France, 2022. Euro Surveill 28:2300095. doi:10.2807/1560-7917.ES.2023.28.42.2300095 37855905 PMC10588306

[14] Yang Y, Liu H, Chen L, Mao M, Zhang X, Zhou L. 2023. Molecular characterization and comparison of *bla*_NDM-1_-carrying and *bla*_NDM-5_-harboring IncX3-type plasmids in carbapenem-resistant *Klebsiella pneumoniae*. Microbiol Spectr 11:e01028-23. doi:10.1128/spectrum.01028-23 37623430 PMC10581223

[15] Aja-Macaya P, Blanco-Martín T, Mateo-León C, Del Rosario-Quintana C, Piña C, de la Cruz-Tabares E, Rodríguez-Pallares S, González-Pinto L, Beceiro A, Oviaño M, Bou G, de Artola DG-M, Arca-Suárez J. 2026. Emergence of New Delhi metallo-β-lactamase 14-producing *Klebsiella pneumoniae* sequence type 147 clone in spain and outbreak in the Canary Islands. Emerg Infect Dis 32:63–73. doi:10.3201/eid3201.251504 41612546 PMC12870054

[16] Le DQ, Nguyen SH, Nguyen TT, Nguyen CH, Ho TH, Vo NS, Nguyen T, Nguyen HA, Cao MD. 2024. AMRViz enables seamless genomics analysis and visualization of antimicrobial resistance. BMC Bioinformatics 25:193. doi:10.1186/s12859-024-05792-9 38755527 PMC11100100

[17] Candela A, Rodríguez-Temporal D, Lumbreras P, Guijarro-Sánchez P, Arroyo MJ, Vázquez F, Beceiro A, Bou G, Muñoz P, Oviaño M, Fernández J, Rodríguez-Sánchez B. 2025. Multicenter evaluation of Fourier transform infrared (FTIR) spectroscopy as a first-line typing tool for carbapenemase-producing *Klebsiella pneumoniae* in clinical settings. J Clin Microbiol 63:e0112224. doi:10.1128/jcm.01122-24 39601577 PMC11784409

[18] Dinkelacker AG, Vogt S, Oberhettinger P, Mauder N, Rau J, Kostrzewa M, Rossen JWA, Autenrieth IB, Peter S, Liese J. 2018. Typing and species identification of clinical *Klebsiella* isolates by Fourier transform infrared spectroscopy and matrix-assisted laser desorption ionization-time of flight mass spectrometry. J Clin Microbiol 56:e00843-18. doi:10.1128/JCM.00843-18 30135233 PMC6204683

[19] Novais Â, Gonçalves AB, Ribeiro TG, Freitas AR, Méndez G, Mancera L, Read A, Alves V, López-Cerero L, Rodríguez-Baño J, Pascual Á, Peixe L. 2024. Development and validation of a quick, automated, and reproducible ATR FT-IR spectroscopy machine-learning model for *Klebsiella pneumoniae* typing. J Clin Microbiol 62:e0121123. doi:10.1128/jcm.01211-23 38284762 PMC10865814

[20] Pathogenwatch. Pathogenwatch: genomic surveillance platform for microbial pathogens. Accessed 05 March 2026. https://pathogen.watch.

[21] Lam MMC, Wick RR, Watts SC, Cerdeira LT, Wyres KL, Holt KE. 2021. A genomic surveillance framework and genotyping tool for *Klebsiella pneumoniae* and its related species complex. Nat Commun 12:4188. doi:10.1038/s41467-021-24448-3 34234121 PMC8263825

[22] Parks DH, Imelfort M, Skennerton CT, Hugenholtz P, Tyson GW. 2015. CheckM: assessing the quality of microbial genomes recovered from isolates, single cells, and metagenomes. Genome Res 25:1043–1055. doi:10.1101/gr.186072.114 25977477 PMC4484387

[23] Seemann T. 2026. Snippy: fast bacterial variant calling from NGS reads. https://github.com/tseemann/snippy.

[24] Price MN, Dehal PS, Arkin AP. 2009. FastTree: computing large minimum evolution trees with profiles instead of a distance matrix. Mol Biol Evol 26:1641–1650. doi:10.1093/molbev/msp077 19377059 PMC2693737

[25] Severiano A, Pinto FR, Ramirez M, Carriço JA. 2011. Adjusted Wallace coefficient as a measure of congruence between typing methods. J Clin Microbiol 49:3997–4000. doi:10.1128/JCM.00624-11 21918028 PMC3209087

[26] Cugmas M. 2026. MRI: multivariate regression with Iterative variable selection. https://cran.r-project.org/package=mri.

[27] Yu G. 2026. ggtree: visualization and annotation of phylogenetic trees. https://bioconductor.org/packages/ggtree.

[28] Wickham H. 2026 ggplot2: elegant graphics for data analysis. https://cran.r-project.org/package=ggplot2

[29] Robertson J, Nash JHE. 2018. MOB-suite: software tools for clustering, reconstruction and typing of plasmids from draft assemblies. https://github.com/phac-nml/mob-suite 10.1099/mgen.0.000206 PMC6159552 30052170

[30] Zheng J, Ge Q, Yan Y, Zhang X, Huang L, Yin Y. 2023. dbCAN3: automated carbohydrate-active enzyme and substrate annotation. https://pro.unl.edu/dbCAN2/. 10.1093/nar/gkad328 PMC10320055 37125649

[31] Martak D, Valot B, Sauget M, Cholley P, Thouverez M, Bertrand X, Hocquet D. 2019. Fourier-transform infrared spectroscopy can quickly type gram-negative bacilli responsible for hospital outbreaks. Front Microbiol 10:1440. doi:10.3389/fmicb.2019.01440 31293559 PMC6606786

[32] Wyres KL, Wick RR, Gorrie C, Jenney A, Follador R, Thomson NR, Holt KE. 2016. Identification of *Klebsiella* capsule synthesis loci from whole genome data. Microb Genom 2:e000102. doi:10.1099/mgen.0.000102 28348840 PMC5359410

[33] Wang-Wang JH, Soler L, Martró E, Clarà G, Hidalgo J, Casas I, García-Quesada MJ, Giménez M, Saludes V, Bordoy AE, Cardona PJ. 2025. Rapid Detection of VREfm Clusters: FTIR spectroscopy as a practical alternative to whole-genome sequencing. Open Forum Infect Dis 12:ofaf718. doi:10.1093/ofid/ofaf718 41377592 PMC12687595

[34] Castillo-Polo JA, Hernández-García M, Maruri-Aransolo A, de la Vega C, Ruiz-Garbajosa P, Cantón R. 2024. Evolution of ceftazidime-avibactam and cefiderocol resistance in ST131-H30R1-*Escherichia coli* isolates with KPC-3 mutants and application of FTIR biotyping. Microbiol Spectr 12:e0277623. doi:10.1128/spectrum.02776-23 38415657 PMC10986490

